# P02-024 - Clinical impact of V198M mutation in NLRP3 gene

**DOI:** 10.1186/1546-0096-11-S1-A131

**Published:** 2013-11-08

**Authors:** R Caorsi, A Insalaco, L Obici, L Cantarini, A Meini, M Alessio, L Lepore, F Zulian, F Caroli, I Ceccherini, A Martini, M Gattorno

**Affiliations:** 1 2nd Division Of Pediatrics, Istituto Gaslini, University of Genoa, Genova, Italy; 2 Department of Pediatrics, Ospedale Pediatrico Bambino Gesù, Roma, Italy; 3 Amyloidosis Research and Treatment Center, IRCCS Fondazione Policlinico San Matteo, Pavia, Italy; 4 Department of Rheumatology, Policlinico Le Scotte, University of Siena, Siena, Italy; 5 Department of Pediatrics, Spedali Civili, Brescia, Italy; 6 Department of Pediatrics, Federico II Hospital, Napoli, Italy; 7 Department of Pediatrics, IRCCS Burlo Garofalo, University of Trieste, Trieste, Italy; 8 Department of Pediatrics, University of Padua, Padova; 9 Department of Genetics, Istituto Gaslini, Genova, Italy

## Introduction

The V198M mutation is described as a possible hypomorphic variant of the NLPR3 gene. However the impact of this mutation is still largely unknown.

## Objectives

To analyse the prevalence of V198M mutation in patients with a clinical history suggestive for CAPS and to describe the clinical and laboratory findings of patients carrying this mutation.

## Methods

From 2002 the molecular analysis of the NPLR3 gene was performed in 524 patients with a clinical history suggestive for CAPS. In order to estimate the pravalence of the mutation of this gene in the healty population 98 healthy individuals were also analyzed for the same mutation.

## Results

The V198M mutation was found in 13 screened patients: 10 were heterozygous for the mutation only. In one patient with a typical MWS phenotype the V198M variant was associated with the Q703K and the D303N mutation of the same gene. In a patient a low-penetrance mutation of TNFRSF1A gene (P46L) was also found, while another one carried the A91V mutation of Pfr1 gene.

Out of the 10 patients heterozygous for the V198M mutation, five displayed a story of periodic fever associated with urticarial rash, arthralgia and transient arthritis, associated with elevation of acute phase reactants and responding to steroid treatment on demand or to treatment with IL-1 blockers. In two patients the clinical picture was mild and uniquely characterized by urticarial rash and arthralgia, often induced by cold, but not associated with elevation of acute phase reactants. The other three patients presented episodes of fever with an inconstant elevation of acute phase reactants and not associated to other symptoms suggestive of CAPS; however one of this patients developed renal amiloidosis. The patients carrying the P46L mutation of TNFRSF1A gene presented periodic fever with athralgia and headache, not associated with elevation of acute phase reactants. The patient carrying the A91V mutation of Pfr1 gene presented some clinical characteristics suggestive of CINCA syndrome associated to not typical ones; this patient died at the age of 4.7 years with a clinical pictures consistent of MAS.

3 patients were treated with IL-1 blockers (anakinra at the staring dosage of 1 mg/kg and canakinumab at the starting dosage of 2 mg/kg every 8 weeks), two heterozygous for V198M and one compound heterozygous for V198M, D303D and Q703 K, with a rapid a complete control of the clinical manifestations.

None of the healthy individuals screened for the V198M mutation turned out to be positive.

## Conclusion

This study confirms the low-penetrance of the V198M mutation of the NLPR3 gene. However a minority of these patients may present a clinical phenotype consisting with a CAPS, thus requiring treatment with IL-1 blockers.

## Disclosure of interest

None declared.

